# Supplementary material for: The influence of common testing floor surfaces on force plate data: Implications for standardisation
Source: PLoS One. 2025 Jul 3;20(7):e0319304. doi: 10.1371/journal.pone.0319304 (PMC12225842; doi:10.1371/journal.pone.0319304)
Supplement: Attachment — Submitted filename: Response to Reviewers.docx [file pone.0319304.s005.docx]

**Response to Reviewers**

Dear Editor,

We sincerely thank you and the reviewers for your thoughtful and constructive feedback on our manuscript, titled *The influence of common testing floor surfaces on force plate data: implications for standardisation*. Below, we provide a point-by-point response to each of the comments raised by the reviewers within the Comments for Authors letter. Additionally comments within the manuscript have been addressed. Changes have been incorporated into the revised manuscript, and we trust these revisions have improved the clarity, structure, and impact of the paper.

**Reviewer Comments and Author Responses**

**Abstract**

**Comment 1:**
*In the abstract, the statement that “there is no research into the effect” might be slightly overstated. It would be more accurate and appropriately cautious to state that “there is limited research,” which acknowledges the broader context while still emphasizing the novelty of the work.*

**Response:**
We agree with this observation and are happy to accept the reviewers revised wording which has been amended by the reviewer directly within the abstract.

**Comment 2:**
*Clarifying early in the abstract that the data were collected using a mechanical testing device, rather than human participants, would improve the reader’s understanding of the experimental model.*

**Response:**
Thank you for this suggestion. The abstract states that a mechanical testing device was used within line 27 of the manuscript. We have included a further statement to highlight that human participants were not used to make the methodology immediately clear to the reader.

**Comment 3:**
*Including brief details on the number of impacts or trials would also strengthen the impression of methodological robustness.*

**Response:**
We have not specifically addressed this within the abstract. This is due to conserving word count and to avoid confusion to the reader as the methods used different number of impacts for the reference and test surfaces, in line with standards. Similarly, the guideline to authors states the abstract should “Explain how the study was done, including any model organisms used, without methodological detail”.

**Introduction**

**Comment 4:**
*The introduction is well-motivated, but paragraphs two and three are dense and could benefit from being broken into shorter sections.*

**Response:**
Thank you for your suggestion, we have restructured paragraphs two and three in the introduction, breaking them into shorter parts for improved readability.

**Comment 5:**
*The distinction between superior and inferior surfaces could be more visually emphasized—perhaps through italics or a parenthetical definition.*

**Response:**
We have now italicised the terms “superior” and “inferior” at their first mention and provided a consistent parenthetical definition to enhance clarity.

**Comment 6:**
*To support the practical context, include at least one additional peer-reviewed source rather than relying on a blog post.*

**Response:**
Thank you, whilst we have left the blog post in, we are happy to remove the sentence outlining the blog post citation if you feel appropriate. This industry led blog post was only included as, to date we are yet to find a peer-reviewed article that discusses the inferior surface-force plate interaction. Similarly, this blog post article was only included as, whilst it is not peer-reviewed, it was authored by a reputable Professor who has authored over 50 peer-reviewed articles within the field of sports biomechanics. We reiterate that we are happy to remove the statement regarding the blog post if you feel appropriate.

**Methods**

**Comment 7:**
*In the Materials and Methods section, clarify how the flooring surfaces were selected—was this based on observational data, informal surveying, or another criterion?*

**Response:**
We have added a sentence explaining that flooring surfaces were selected based on informal discussions with associates who have experience within professional sporting training facilities and the floorings used.

**Comment 8:**
*Cite Hopkins (2002) more formally in a complete sentence when presenting ICC thresholds.*

**Response:**
Thank you, Hopkins (2002) has been more formally cited in a complete sentence and cited according to correct referencing style.

**Comment 9:**
*Introduce the exact mass of the mechanical device and specific model versions of the force plates earlier in the Materials and Methods section.*

**Response:**
Thank you for your suggestion. We agree that providing precise details such as the mechanical device’s mass and the specific model versions of the force plates early in the methods section is essential for reproducibility. While these details are currently included under the second subheading of the Materials and Methods section, we chose to introduce the flooring types under the first subheading, as the primary focus of the study is the effect of inferior surface conditions. Introducing the force plate specifications and mechanical device parameters in the subsequent subheading allows us to maintain a logical flow from the experimental context to the testing setup. Whilst we would prefer the current structure, if you would prefer the mechanical device and force plate details earlier, we could move the subheading order to be (1) Mechanical testing device, (2) Mechanical testing protocol, (3) Flooring conditions, (4) Data analysis, (5) Statistical analyses. Please advise your preference if necessary.

**Comment 10:**
*Provide a brief rationale for the decision to use three impacts per surface.*

**Response:**
A brief rationale has been added to justify the choice of three impacts per surface, referencing prior methodological standards (EN:14808) and pilot testing feasibility.

**Results**

**Comment 11:**
*The narrative of the pairwise comparisons in the results section could be streamlined. Focus more on patterns and implications.*

**Response:**
Thank you for this suggestion. We have revised the results narrative to streamline and reduce redundancy. Specifically, we have removed some of the full listing of individual p-values and effect sizes for each pairwise comparison from the main text. Instead, we have summarised the key significant differences across surface conditions and removed narrative on the non-significant differences. This revision enhances the clarity and conciseness of the results section.

**Comment 12:**
*In tables, bold statistically significant results to aid reader scanning.*

**Response:**
Thank you for your suggestion. Due to the complexity of highlighting significant differences between multiple flooring conditions, we have opted not to bold within tables. Significant differences are however visually presented within figures 2 and 3 and this outlined within the written results summary.

**Comment 13:**
*Expand figure legends so they can be understood independently of the main text.*

**Response:**
Figure legends have been revised to include more context, enabling them to stand alone more effectively.

**Comment 14:**
*Ensure color schemes are accessible for readers with color vision deficiencies and clarify whether boxplots show mean or median values.*

**Response:**
Thank you for your comment. We acknowledge the importance of ensuring accessibility for readers with colour vision deficiencies (CVD). While the current heatmap uses a red–blue colour scheme, which may not be ideal for some readers with CVD, we have addressed accessibility in the following ways:

- All heatmap cells include overlaid numeric values, allowing precise interpretation regardless of colour perception.
- The colour gradients serve only as a visual aid and are not the sole means of conveying information.

Additionally, we have clarified in the Fig 2 caption that the boxplots represent median values, as well as providing further descriptives to explain the meaning of the box, whiskers and dots. We hope this ensures greater clarity for the reader.

**Discussion**

**Comment 15:**
*Reword repetitive instances of “Practitioners should exert caution...” for improved readability.*

**Response:**
Thank you for highlighting the repetitive instances. We have rephrased the first instance to state “The findings of the present study therefore highlight the importance of carefully considering the testing location and the associated flooring surface, when conducting force plate assessments.” We feel that this maintains the message conveyed whilst avoiding repetition.

**Comment 16:**
*Expand the explanation regarding the CarpetG anomaly. Could a pilot test or durability assessment clarify this unexpected result?*

**Response:**
We have expanded the discussion of the CarpetG anomaly to outline that in retrospect a preliminary material property assessment should have been conducted to provide insight as to whether the carpet location was appropriate for inclusion. A suggestion for future pilot material property assessment has also been emphasised.

**Comment 17:**
*Include a brief reflection on the cost-benefit considerations of standardizing flooring surfaces in practical environments.*

**Response:**
Thank you for your suggestion. A paragraph has been added highlighting the cost-benefit trade-offs of standardising floor conditions in real-world testing, with reference to both research and practice constraints. Whilst you have suggested this should occur within the Discussion section; we have placed this paragraph within the Conclusion and practical considerations section.

**Comment 18:**
*Improve the visibility of the limitations section, either via a clear subtitle or formatting.*

**Response:**
We have now included a dedicated subheading for the limitations section. This is structured to allow clearer visibility to readers.

**Comment 19:**
*Add a comment on the generalizability of findings to other mechanical testing devices or force plate brands.*

**Response:**
We have included a paragraph to the limitations section noting that results may vary with different mechanical testing devices or force plate models, affecting generalisability.

**Comment 20:**
*Consider ending the conclusion with a forward-looking sentence encouraging future research into standardization across broader environments.*

**Response:**
We have added a closing sentence to the conclusion and practical considerations section, encouraging future studies to investigate standardisation practices across a wider range of testing environments.

We appreciate the reviewer’s and editor’s time and insightful suggestions. We believe that these changes have significantly improved the clarity and overall quality of our manuscript.

Sincerely,
Laura Smith
